# Complete Genome Sequence of *Listeria monocytogenes* LL195, a Serotype 4b Strain from the 1983–1987 Listeriosis Epidemic in Switzerland

**DOI:** 10.1128/genomeA.00152-12

**Published:** 2013-01-31

**Authors:** Thomas Weinmaier, Martin Riesing, Thomas Rattei, Jacques Bille, Carolina Arguedas-Villa, Roger Stephan, Taurai Tasara

**Affiliations:** a Department of Computational Systems Biology, Faculty of Life Sciences, University of Vienna, Vienna, Austria; b Institute of Microbiology, University of Lausanne, Lausanne, Zurich, Switzerland; c Institute of Food Safety and Hygiene, Vetsuisse Faculty, University of Zurich, Zurich, Switzerland

## Abstract

The complete genome sequence of *Listeria monocytogenes* LL195, a serotype 4b clinical strain isolated during the 1983–1987 listeriosis epidemic in Switzerland, is presented.

## GENOME ANNOUNCEMENT

*Listeria monocytogenes* is a Gram-positive opportunistic foodborne bacterial pathogen. It causes listeriosis, an illness with serious consequences for immunocompromised people, such as pregnant women, neonates, and elderly people (1). Switzerland experienced a long-lasting listeriosis epidemic from 1983 to 1987, which was associated with the consumption of Vacherin Montd’or cheese and led to 122 clinical cases and 31 fatalities (2, 3). We determined the complete genome sequence of *L. monocytogenes* LL195, a serotype 4b strain recovered from a listeriosis case during this epidemic.

A paired-end library of the LL195 genome was created and sequenced using the Illumina Genome Analyzer (GA) II sequencer. In total, 107,606,904 reads of 50 bp were obtained from the sequencing (~1,700-fold genome coverage). The genome of *L. monocytogenes* F2365, a serotype 4b soft-cheese-derived isolate from the 1985 California Jalisco cheese listeriosis outbreak, was used as a reference (4, 5) for the comparative assembler AMOScmp (6). A total of 16 contigs were produced in the assembly. All gaps were closed using the Sanger sequencing method, resulting in one unambiguous scaffold. This single scaffold represents the complete circular LL195 chromosome, consisting of 2,904,662 bp with a G+C content of 38%.

The coding sequences (CDSs) were predicted based on a house-internal workflow that integrates *ab initio* predictions from Glimmer, GeneMark (7), Prodigal (8), and Critica (9) with homology information derived from a BLASTp search against the NCBI nonredundant (NR) database (10). Noncoding RNAs were identified using tRNAscanSE (11) and RNAmmer (12) and by searching against the Rfam database (13). Functional annotation of the CDSs was based on InterProScan (14) and the Swiss-Prot and trEMBL (15) databases.

The genome sequence of LL195 contains 2,838 protein-coding genes, six 16S-5S-23S operons, and 67 tRNA genes, and it harbors no plasmid or prophage genes. Although LL195 and F2365 genomes share high levels of similarity (99.9%), the LL195 genome has 197 single nucleotide polymorphisms (SNPs) and 37 insertions and deletions compared against the F2365 genome. Although virulence genes are highly conserved between the two genomes, there are single SNPs in *hly* and *inlB* genes, as well as three SNPs and a single nucleotide deletion in the *inlJ* gene in LL195 compared against F2365. Notably, although the F2365 *inlB* is a pseudogene, due to a nonsense mutation that creates a premature stop codon (16), an SNP in the LL195 genome leads to an intact *inlB* gene in this strain.

### Nucleotide sequence accession number.

The sequence and annotation of the *L. monocytogenes* LL195 genome have been deposited in the EMBL database under the accession no. HF558398.
